# Clinical Utility of the Modified Pulmonary Index Score as an Objective Assessment Tool for Acute Asthma Exacerbation in Children

**DOI:** 10.31662/jmaj.2018-0010

**Published:** 2018-09-28

**Authors:** Takanobu Maekawa, Yukihiro Ohya, Masashi Mikami, Satoko Uematsu, Akira Ishiguro

**Affiliations:** 1 Division of Pediatrics, Department of General Pediatrics and Interdisciplinary Medicine, National Center for Child Health and Development, Tokyo, Japan; 2 Division of Allergy, Department of Medical Subspecialties, National Center for Child Health and Development, Tokyo, Japan; 3 Division of Biostatistics, Center for Clinical Research, National Center for Child Health and Development, Tokyo, Japan; 4 Division of Emergency Service and Transport Medicine, Department of General Pediatrics and Interdisciplinary Medicine, National Center for Child Health and Development, Tokyo, Japan; 5 Department of Postgraduate Education and Training, National Center for Child Health and Development, Tokyo, Japan

**Keywords:** Asthma, child, emergency department, modified pulmonary index score, validity

## Abstract

**Introduction:**

The Modified Pulmonary Index Score (MPIS) was developed as an objective assessment tool for acute asthma exacerbation in children. Although it is considered reliable, there are no known studies of its clinical utility. The objective of this study was to evaluate the validity of the MPIS for children with acute asthma in a clinical setting.

**Methods:**

In this retrospective study conducted between July 2009 and June 2011 using electronic medical records at the emergency department of a single pediatric medical center in Tokyo, Japan, the MPIS was recorded for patients with acute asthma at initial assessment and after treatment with an inhaled beta-agonist. We evaluated the responsiveness and predictive validity of the MPIS using disposition as an outcome.

**Results:**

A total of 2242 patients were assessed using the MPIS (median age, 3 years; 71.2% patients were 5 years or younger). The mean (SD) MPIS at initial assessment was 7.1 (3.6) and was significantly higher for the admission group than for the non-admission group (9.9 [2.9] vs. 5.9 [3.1]; *P* < 0.001). The receiver operator characteristic curve of the initial MPIS for hospital admission demonstrated moderate predictive ability (area under the curve, 0.83). An MPIS reduction of 3 or more indicated a clinically significant change when the MPIS at initial assessment was between 6 and 10 (risk ratio for admission [95% CI], 0.41 [0.28–0.60]; *P* < 0.001).

**Conclusion:**

The MPIS demonstrated good concurrent validity, predictive validity, and responsiveness in a wide range of clinical settings.

## Introduction

Bronchial asthma is one of the most common chronic childhood disorders worldwide. Every day and night, many children visit the emergency department (ED) seeking medical treatment for acute asthma exacerbations, and physicians provide treatment based on their assessments of acute asthma severity. Appropriate objective assessment of acute asthma severity is essential for planning treatment, evaluating the response to the treatment, and sharing patient information among medical professionals. Conventionally, a four-grade severity classification (mild, moderate, or severe exacerbations and respiratory failure) was recommended as an initial assessment in guidelines, including the Global Initiative for Asthma (GINA) ^(1)^, British guidelines on the management of asthma ^(2)^, the Expert Panel Report 3 (EPR3) in the United States ^(3)^, and the Japanese pediatric guidelines for the treatment and management of bronchial asthma (JPGL) ^(4)^. Although this simple classification facilitates prompt assessment of severity and ensures appropriate initiation of treatment without delay, it is difficult to represent the wide distribution of severity or detect a small clinical change. Accordingly, several clinical symptom scoring systems aiming to assess asthma severity have been developed and evaluated. The Modified Pulmonary Index Score (MPIS) was one of the clinical scores developed by Carroll et al. in 2005 to assess the severity of acute asthma exacerbation in children ^(5)^ and was applied as a primary outcome measure in some clinical trials ^(6), (7), (8)^. The MPIS was previously reported to have 1) good inter-rater reliability and predictive value for 30 children with a mean age of 7.6 years ^(5)^, 2) good internal consistency and inter-rater reliability for 25 children, including 13 preschool children ^(9)^; and 3) good predictive value for hospital admission as an outcome ^(10), (11)^. However, its utility for a larger population, including preschool children, has not been fully studied.

In this study, we evaluated the predictive validity and clinical utility of the MPIS in a pediatric population, including preschool children.

## Materials and Methods

This retrospective study used electronic medical records from the ED of a single pediatric medical center in Tokyo, Japan. This study was approved by the Institutional Ethics Committee of the National Center for Child Health and Development, Setagaya, Tokyo, Japan on 15 June, 2011 (approved code: 679).

The MPIS comprises six items: (1) oxygen saturation on room air (SpO_2_), (2) accessory muscle use, (3) inspiratory-to-expiratory flow ratio (I:E ratio), (4) degree of wheezing, (5) heart rate (HR), and (6) respiratory rate (RR). Each item was rated using a score of 0 to 3 based on severity. The range of the total score is 0–18. An increase in the total score indicates an increase in severity (Table 1).

**Table 1.** The Modified Pulmonary Index Score (MPIS).

		Score			
		0	1	2	3
Oxygen saturation, %		>95	93–95	90–92	<90
Accessory muscle use		None	Mild	Moderate	Severe
Inspiratory-to-expiratory flow ratio		2:1	1:1	1:2	1:3
Wheezing		None	End expiratory	Inspiratory and expiratory wheeze, good aeration	Inspiratory and expiratory wheeze, decreased aeration
Heart rate, (/min)					
	<3 years old	<120	120–140	141–160	>160
	≥3 years old	<100	100–120	121–140	>140
Respiratory rate, (/min)					
	<6 years old	≤30	31–45	46–60	>60
	≥6 years old	≤20	21–35	36–50	>50

In 2008, we introduced the MPIS as a standard assessment tool for children presenting with acute asthma exacerbation at our pediatric ED. We created a custom template in the electrical medical record (EMR) system to promote MPIS use. In this template, the MPIS was automatically calculated and recorded on the EMR when physicians entered the (1) SpO_2_, (5) HR, and (6) RR and selected a score of 0 to 3 for the other three items, i.e., (2) accessory muscle use, (3) I:E ratio, and (4) degree of wheezing. Along with the MPIS, a conventional four-grade severity classification of asthma exacerbations (mild, moderate, or severe exacerbations and respiratory failure) according to JPGL was required to be assessed. All data fields, including MPIS and JPGL, should be complete before submission to the EMR.

The ED physicians were trained using a MPIS computer tutorial that contained recorded auscultation sounds, video images, and computer graphics of asthma exacerbation of varying severity and were encouraged to use the MPIS to assess asthma exacerbation.

### Data collection

We extracted the clinical data of the patients who 1) visited the ED between July 2009 and June 2011, 2) were aged 1–18 years, 3) were diagnosed with acute asthma exacerbation, and 4) were assessed using the MPIS. Patients who 1) had cyanotic congenital heart disease, 2) had a tracheotomy, and 3) received home oxygen therapy were excluded from analysis. The following clinical data were collected: age, gender, MPIS score (at presentation and after initial treatment), asthma exacerbation severity classification (mild, moderate, or severe exacerbations and respiratory failure) according to JPGL ^(4)^, treatments provided in the ED, and final outcome (non-admission, hospital admission, or pediatric intensive care unit [PICU] admission). For patients who required hospital or PICU admission, we reviewed the medical treatments provided during their hospital stay, length of oxygen inhalation, and length of hospital stay. Continuous inhalation therapy was performed using *l*-isoproterenol, which is widely used for severe asthma exacerbations in Japan ^(12)^.

### Statistical analysis

The features of the patients were descriptively analyzed. Concurrent validity was evaluated by comparing the MPIS distributions with the four severity classifications of the JPGL using the Tukey–Kramer multiple comparisons test. The relationship between the MPIS and outcomes was evaluated using the Student’s *t*-test and correlation coefficient. To assess predictive validity, we evaluated the correlation of the MPIS at initial assessment and the outcome (non-admission, hospital admission, or PICU admission) using receiver operator characteristic curve analysis. Responsiveness was evaluated nonparametrically by the correlation between the MPIS reduction (defined as the MPIS at initial assessment minus the MPIS at additional assessment after treatment) and the outcome using the Wilcoxon rank sum test. Analyses were performed for all age groups, including a younger age group comprising 1- to 5-year-old patients and an older age group comprising 6- to 18-year-old patients. Data were analyzed using SAS software, version 9.4 (SAS Institute Inc.).

## Results

**Figure 1.**
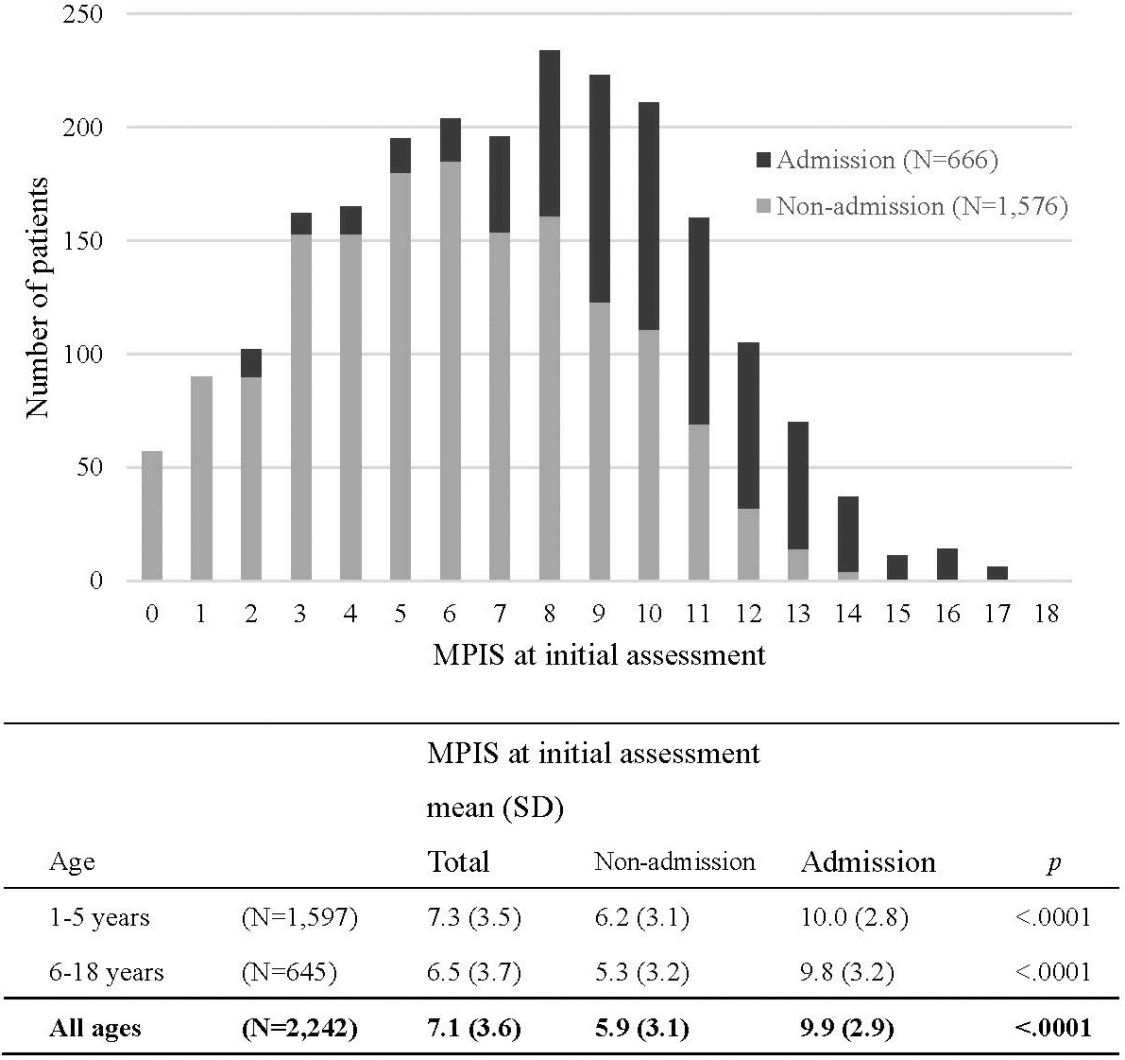
Distribution of the MPIS at initial assessment and patient dispositions.

A total of 2242 patients were assessed using the MPIS (median age, 3 years; 71.2% of patients were 5 years or younger) from the 2669 patients who visited the ED with acute asthma exacerbation during the study period. Of the 2242 patients, 666 (29.7%) required hospital admission. The study population characteristics are summarized in Table 2. At initial assessment, 416 (18.6%) patients had a fever ≥ 38.0 °C, and the rate was higher in the younger age group. Among 666 admitted patients, 29 patients required continuous inhalation with *l*-isoproterenol, and 8 patients required mechanical ventilatory support with intratracheal intubation. The distribution of the MPIS at initial assessment and their outcomes is shown in Figure 1. The mean (SD) MPIS at initial assessment was 7.1 (3.6) and was significantly higher in the admission group than in the non-admission group (9.9 [2.9] vs. 5.9 [3.1]; *P* < 0.001). The results were the same for both the younger and older subgroups.

**Table 2.** Study Population Characteristics.

		All ages (N = 2,242)	1–5 years (N = 1,597)	6–18 years (N = 645)
Age (median [IQR])		3 (2–6)	2 (1–3)	8 (7–10)
Gender (M:F)		53:47	51:49	58:42
Body temperature ≥ 38.0°C at initial assessment (N [%])		416 (18.6)	341 (21.4)	75 (11.6)
MPIS at initial assessment (mean [SD])		7.1 (3.6)	7.3 (3.5)	6.5 (3.7)
Severity of asthma (JPGL) (N [%])				
	Mild	862 (38.4)	591 (37.0)	271 (42.0)
	Moderate	1,129 (50.4)	834 (52.2)	295 (45.7)
	Severe	242 (10.8)	164 (10.2)	78 (12.1)
	Respiratory failure	9 (0.4)	8 (0.5)	1 (0.2)
Disposition (N [%])				
	Non-admission	1,576 (70.3)	1,103 (69.1)	473 (73.3)
	Hospital admission	666 (29.7)	494 (30.9)	172 (26.7)
	PICU admission	8 (1.2)	5 (1.0)	3 (1.7)
Outcomes after hospital admission				
	Continuous inhalation required (N [%])	29 (4.3)	17 (3.4)	12 (7.0)
	Ventilator support required (N [%])	8 (1.2)	5 (1.0)	3 (1.7)
	Length of oxygen inhalation in days (median [IQR])	3 (2–5)	3 (2–4)	3 (2–5)
	Length of hospital stay in days (median [IQR])	5 (4–9)	5 (4–9)	6 (4–11)

IQR: interquartile range

### Concurrent validity

**Figure 2.**
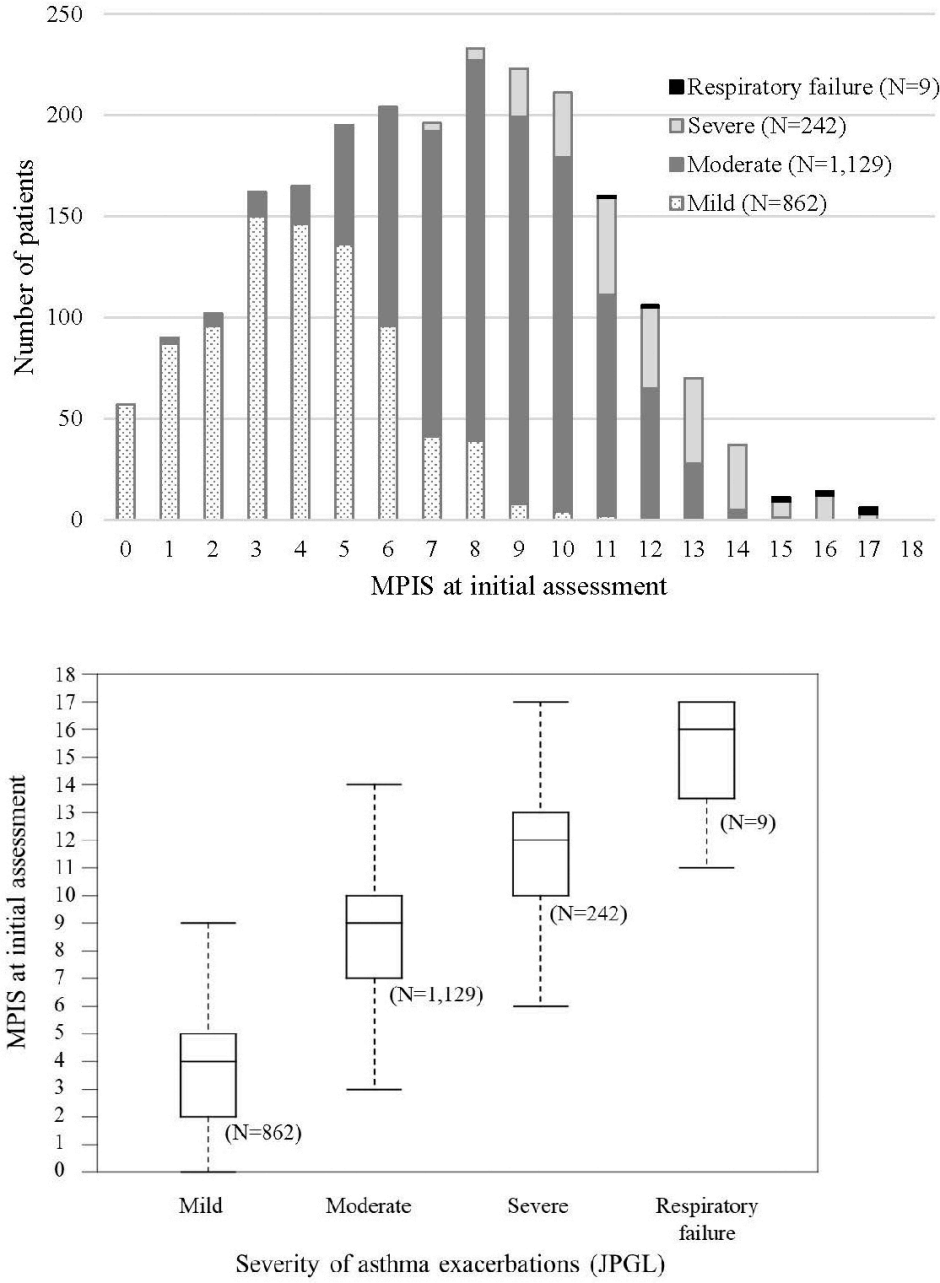
Correspondence of the MPIS to severity of asthma exacerbations (JPGL). The whiskers of the box plot are drawn to the furthest point within 1.5 × IQR from the box. The distributions of the MPIS were significantly different between groups (Kruskal–Wallis rank sum test, *P* < .0001).

The MPIS distributions for each of the four JPGL severity classifications are shown in Figure 2. The median MPIS (interquartile range) was 4 (2–5), 9 (7–10), 12 (10–13), and 16 (13.5–17) for mild, moderate, and severe exacerbation, and respiratory failure, respectively. These distributions differed significantly for each class as determined by the Tukey–Kramer multiple comparison test (*P* < 0.001).

### Predictive validity

The mean (SD) MPIS at initial assessment was 7.1 (3.6) and was significantly higher for the admission group than for the non-admission group (9.9 [2.9] vs. 5.9 [3.1]; *P* < 0.001). Along with the MPIS, a higher score for all six items, younger age class, and body temperature ≥ 38.0 °C at initial assessment were significant predictors of hospital admission. After adjustment for these variables, a higher score for all six items and higher body temperature were the independent predictors. The adjusted odds ratios are presented in Table 3.

**Table 3.** Adjusted Risk of Hospital Admission at Initial Assessment.

		Odds Ratio	95% Confidential Interval	*p*
Age class: 1–5 years		1.00	0.78–1.30	0.981
Body temperature: ≥38.0		1.59	1.21–2.08	<0.001
MPIS items: Score ≥2				
	Oxygen saturation	4.20	3.07–5.75	<0.001
	Accessory muscle use	2.05	1.60–2.63	<0.001
	Inspiratory-to-expiratory flow ratio	1.59	1.25–2.03	<0.001
	Wheezing	2.23	1.75-2.83	<0.001
	Heart rate	1.95	1.51–2.51	<0.001
	Respiratory rate	2.41	1.91–3.05	<0.001

The statistical values of the MPIS to predict hospital admission, stratified by age classes and body temperature, are summarized in Table 4. The receiver operator characteristic curve of the initial MPIS for hospital admission demonstrated moderate predictive ability (area under the curve [AUC], 0.83). The cutoff value of the MPIS for all patients was 8 or higher, with sensitivity and specificity of 83.6% and 67.4%, respectively. The cutoff values were higher for the younger age class and for patients with a fever ≥ 38.0 °C.

**Table 4.** Predictive Validity of MPIS.

Age	BT	MPIS	Sens	Spec	LR+	LR−	PPV	NPV	Sens + Spec		AUC
**All ages**											
	**All**	≥12	0.291	0.968	9.182	0.732	0.795	0.764	1.260		**0.825**
		≥11	0.428	0.924	5.667	0.619	0.705	0.793	1.352		**(0.807–0.844)**
		≥10	0.578	0.854	3.961	0.494	0.626	0.827	1.432		
		≥9	0.728	0.776	3.251	0.350	0.579	0.871	1.504		
		≥8	0.836	0.674	2.564	0.243	0.520	0.907	1.510	*	
		≥7	0.899	0.576	2.122	0.175	0.473	0.931	1.476		
	≥38	≥12	0.350	0.953	7.408	0.682	0.853	0.651	1.303		0.830
		≥11	0.530	0.906	5.614	0.519	0.815	0.710	1.436		(0.791–0.869)
		≥10	0.672	0.845	4.350	0.388	0.774	0.767	1.518	*	
		≥9	0.781	0.708	2.678	0.309	0.678	0.805	1.490		
		≥8	0.885	0.549	1.964	0.209	0.607	0.859	1.435		
		≥7	0.951	0.412	1.617	0.119	0.559	0.914	1.363		
	<38	≥12	0.269	0.971	9.268	0.753	0.769	0.787	1.240		0.817
		≥11	0.389	0.928	5.389	0.658	0.660	0.809	1.317		(0.796–0.840)
		≥10	0.542	0.856	3.755	0.535	0.575	0.839	1.398		
		≥9	0.708	0.788	3.337	0.371	0.545	0.882	1.496		
		≥8	0.818	0.695	2.685	0.262	0.491	0.914	1.513	*	
		≥7	0.880	0.605	2.225	0.199	0.445	0.933	1.485		
1–5 years											
	All	≥12	0.279	0.967	8.559	0.745	0.793	0.750	1.247		0.820
		≥11	0.427	0.924	5.609	0.620	0.715	0.783	1.351		(0.799–0.842)
		≥10	0.595	0.843	3.794	0.480	0.630	0.823	1.438		
		≥9	0.737	0.755	3.010	0.348	0.574	0.865	1.492	*	
		≥8	0.844	0.643	2.363	0.242	0.514	0.902	1.487		
		≥7	0.903	0.542	1.972	0.179	0.469	0.926	1.445		
	≥38	≥12	0.342	0.947	6.466	0.695	0.839	0.642	1.289		0.812
		≥11	0.533	0.894	5.036	0.522	0.802	0.704	1.427		(0.768–0.857)
		≥10	0.664	0.825	3.806	0.407	0.754	0.754	1.490	*	
		≥9	0.770	0.672	2.346	0.343	0.654	0.784	1.442		
		≥8	0.882	0.508	1.792	0.233	0.590	0.842	1.390		
		≥7	0.954	0.376	1.528	0.123	0.551	0.910	1.330		
	<38	≥12	0.251	0.972	8.840	0.770	0.768	0.776	1.223		0.814
		≥11	0.380	0.930	5.429	0.667	0.670	0.800	1.310		(0.789–0.840)
		≥10	0.564	0.847	3.684	0.514	0.580	0.839	1.411		
		≥9	0.722	0.772	3.174	0.360	0.543	0.881	1.495		
		≥8	0.827	0.671	2.513	0.257	0.485	0.912	1.498	*	
		≥7	0.880	0.577	2.079	0.208	0.438	0.928	1.457		
6–18 years											
	All	≥12	0.326	0.970	11.000	0.695	0.800	0.798	1.296		0.834
		≥11	0.430	0.926	5.814	0.615	0.679	0.817	1.356		(0.798–0.870)
		≥10	0.529	0.879	4.390	0.535	0.615	0.837	1.409		
		≥9	0.703	0.825	4.009	0.360	0.593	0.884	1.528		
		≥8	0.814	0.746	3.208	0.249	0.538	0.917	1.560	*	
		≥7	0.890	0.655	2.581	0.169	0.484	0.942	1.545		
	≥38	≥12	0.387	0.977	17.032	0.627	0.923	0.694	1.364		0.895
		≥11	0.516	0.955	11.355	0.507	0.889	0.737	1.471		(0.814–0.976)
		≥10	0.710	0.932	10.409	0.312	0.880	0.820	1.641		
		≥9	0.839	0.864	6.151	0.187	0.813	0.884	1.702	*	
		≥8	0.903	0.727	3.312	0.133	0.700	0.914	1.630		
		≥7	0.935	0.568	2.166	0.114	0.604	0.926	1.504		
	<38	≥12	0.312	0.970	10.298	0.709	0.772	0.811	1.282		0.823
		≥11	0.411	0.923	5.348	0.638	0.637	0.827	1.334		(0.783–0.863)
		≥10	0.489	0.874	3.888	0.584	0.561	0.839	1.363		
		≥9	0.674	0.821	3.754	0.398	0.552	0.884	1.494		
		≥8	0.794	0.748	3.155	0.275	0.509	0.917	1.543		
		≥7	0.879	0.664	2.620	0.181	0.463	0.944	1.544	*	

**Abbreviations:** BT, body temperature in Celsius; MPIS, modified Pulmonary Index Score; Sens, sensitivity; Spec, specificity; LR+, positive likelihood ratio; LR−, negative likelihood ratio; PPV, positive predictive value; NPV, negative predictive value; AUC, area under the curve (95% confidence interval). * Cut off value of each groups stratified by the age classes and the body temperature.

The AUCs were also calculated for the MPIS with deletion of one item to evaluate the contribution of each item to the total score. The resulting AUCs were 0.80, 0.82, 0.82, 0.82, 0.81, and 0.82 for the MPIS without SpO_2_, accessory muscle use, I:E ratio, degree of wheezing, HR, and RR, respectively.

### Responsiveness

**Figure 3.**
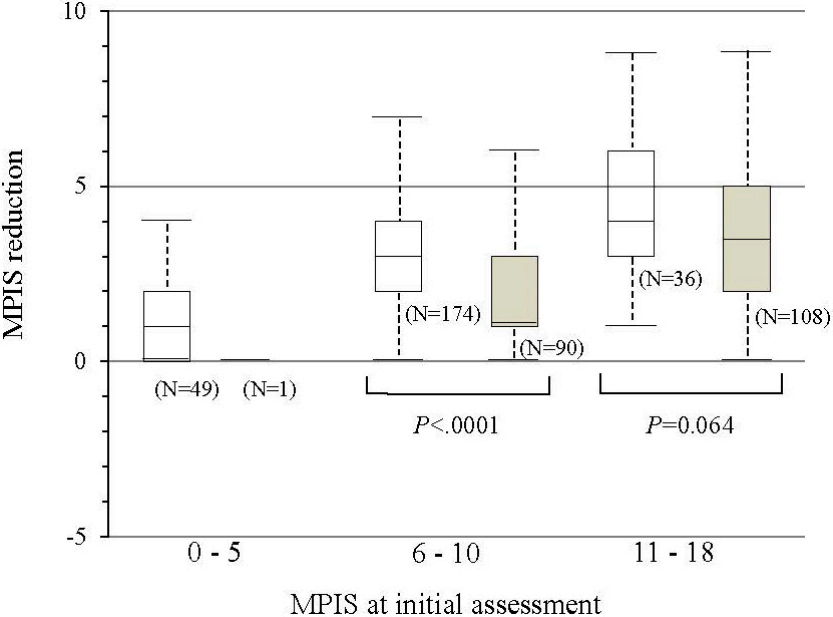
Correlation between the MPIS reduction and hospital admission. The box plot comparing the distribution of the MPIS reduction between the non-admission group (white box) and admission group (gray box), stratified by the MPIS at initial assessment. The MPIS reduction was significantly larger for the non-admission group when the MPIS at initial assessment was between 6 and 10 (Wilcoxon rank sum test, *P* < 0.001).

According to our analyses of the 458 patients who received additional assessments after initial treatment, 448 (97.8%) patients were treated with salbutamol inhalation more than once, and 316 (69.0%) received systemic steroids. The mean MPIS (SD) after treatment was significantly lower than that after initial assessment (9.1 [2.9] vs. 6.2 [2.9]; *P* < 0.001) and was nearly identical for all six items of the MPIS. When the non-admission group was compared with the admission group, the MPIS reduction was significantly larger for the non-admission group when the MPIS at the initial assessment was between 6 and 10 (i.e., moderate asthma exacerbation) (median, 3.0 vs. 1.0; Wilcoxon rank sum test, *P* < 0.001), although the difference was not significant for groups with more severe asthma exacerbation (median, 4.0 vs. 3.5; Wilcoxon rank sum test, *P * < 0.064) (Figure 3). The risk of admission was significantly lower when the MPIS reduction was 3 or more in patients whose MPIS at initial assessment was between 6 and 10 (risk ratio [95% C.I.] = 0.41 [0.28–0.60]; *P* < 0001).

## Discussion

In clinical practice, an appropriate objective assessment tool for asthma in children is required. Without a standard tool for assessing acute asthma severity, several clinical asthma scores have been developed and tested ^(13), (14), (15), (16), (17), (18), (19)^. Most of these scores involve signs and symptoms related to asthma severity. Although no single assessment tool appears to be the best, the MPIS has several advantages as follows: it 1) contains three fundamental vital signs, i.e., HR, RR, and SpO_2_, with which ED medical staff are generally familiar; 2) has consecutive scores from 0 to 18 that reflect the distribution of different degrees of severity of acute asthma; and 3) has continuous characteristics capable of reflecting small clinical changes as changes in the score.

This study aimed to evaluate the usefulness of the MPIS in a wide variety of clinical settings by assessing its concurrent validity, predictive validity, and responsiveness.

In this study, of the 2669 patients who visited the ED with acute asthma exacerbation during the study period (2009–2011), 2242 patients (84%) were assessed using the MPIS. The MPIS distribution showed a continuous curve from mild to severe asthma exacerbation in both the total population and hospital admission group, whereas the mean MPIS was higher for the admission group. Although the study was conducted at a single institution, the large subject pool, high coverage rate, and proportional distribution of severity make the results of this study generalizable in terms of evaluating the MPIS’ utility in a real clinical setting.

In the clinical assessment of acute asthma exacerbation, the guidelines recommend classifying severity as mild, moderate, severe, or life-threatening based on the combination of clinical symptoms and vital signs ^(1), (2), (3), (4)^. Even though the threshold of the severity classes varies across guidelines (Table 5), each of the six MPIS items is included in the severity assessment. In this study, the MPIS distribution differed significantly for each JPGL severity level, indicating good concurrent validity of the MPIS. The MPIS showed a good overlap with the JPGL classifications of mild (0–5), moderate (6–10), and severe exacerbations (11–15), and respiratory failure (16–18). This overlap can help medical staff translate the MPIS into a conservative assessment of acute asthma.

**Table 5.** Severity Assessment of Acute Asthma Exacerbations among Guidelines.

	GINA		BTS/SIGN		EPR3	JPGL
	1–5 years	>5 years	1–5 years	>5 years		
**SpO_2_ (%)**						
Mild	>95				>95	>95
Moderate	92–95		≥92		90–95	92–95
Severe	<92		<92		<90	<92
Life threatening						<90
**Heart rate (/minute)**						
Mild	≤200 (0–3 years) ≤180 (4–5 years)	100–120			<100	
Moderate			≤140	≤125	100–120	
Severe	>200 (0–3 years) >180 (4–5 years)	>120	>140	>125	>120	
Life threatening					Bradycardia	
**Respiratory rate (/minute)**						
Mild		Increased			Increased	Slightly increased
Moderate			≤40	≤30	Increased	Increased
Severe		>30	>40	>30	Frequently >30	Increased
Life threatening						Undetermined
**Retraction, accessory muscle use**						
Mild		Not used			Not usually	None-mild
Moderate					Commonly	Apparent
Severe	Marked	In use	Poor respiratory effort		Usually	Marked
Life threatening					Paradoxical thoracoabdominal movement	Marked
**Auscultation**						
Mild	Variable				Moderate, often only end-expiratory wheeze	Mild wheeze
Moderate					Loud; throughout exhalation wheeze	Apparent wheeze
Severe	may be quiet				Usually loud; throughout inhalation and exhalation wheeze	Marked wheeze
Life threatening	Silent chest		Silent chest		Absence of wheeze	Reduced or eliminated
**Daily life**						
Mild	Talks in sentences				Talks in sentences Breathless while walking Can lie down	Talks in sentences, Normal feeding, Can sleep
Moderate	Talks in sentences		Talks in sentences		Talks in phrases Breathless while at rest Difficulty feeding Prefers sitting	Talks in phrases, Difficulty feeding, Occasionally wakes up
Severe	Talks in words or enable to drink		Cannot complete sentences in one breath or too breathless to talk or feed		Talks in words Breathless while at rest Stop feeding, sits upright	Talks in words, Difficulty feeding, Disturbed sleep
Life threatening	Unable to talk		Exhaustion			Impossible to talk Impossible to feed Disturbed sleep
**Altered consciousness**						
Mild	No altered consciousness				May be agitated	No altered consciousness
Moderate	Agitated				Usually agitated	Slightly excited No altered consciousness
Severe	Agitated, confused, or drowsy				Usually agitated	Excited Slightly lowered consciousness
Life threatening	Drowsy or confused		Confused		Drowsy or confused	Confused Lowered consciousness
**PEF (% predicted or % personal best)**						
Mild		>50			≥70	≥60
Moderate			≥50		40–69	30–60
Severe		≤50	33–50		<40	<30
Life threatening			<33		<25	Unmeasurable

GINA: Global Initiative for Asthma; GINA Report, Global Strategy for Asthma Management and Prevention BTS/SIGN: British Thoracic Society/Scottish Intercollegiate Guidelines Network; British guideline on the management of asthma EPR3: Expert Panel Report 3; Guidelines for the Diagnosis and Management of Asthma, National Heart, Lung, and Blood Institute JPGL: Japanese Pediatric Guideline for the Treatment and Management of Asthma

The MPIS also showed moderate predictive validity and responsiveness. In terms of predictive validity, the AUC of the initial MPIS for predicting hospital admission was 0.83, indicating moderate performance in predicting outcome. The AUC was higher for the MPIS with six items than for the MPIS with five items, indicating that the MPIS shows the best performance when all six items are included and each item contributes equally to the scoring system. In terms of responsiveness, a larger MPIS reduction was related to the outcome of non-admission and was significant when the severity of the acute asthma at initial assessment was moderate (i.e., the initial MPIS was between 6 and 10) but was not statistically significant for severe acute asthma (i.e., the initial MPIS was ≥11). Physicians tend to prefer hospital admission when the degree of acute asthma exacerbation at initial presentation is severe even if good clinical improvement is observed after the initial treatment. An MPIS reduction of 3 or more would be a clinically significant change if the patient had moderate asthma exacerbation at initial presentation.

There are several limitations to this study. The first involves the retrospective model of the study. The study population comprised patients with acute asthma clinically diagnosed by physicians without any explicit criteria; therefore, part of the study population may have had viral bronchitis or pneumonia, including respiratory syncytial virus infection, particularly in children aged ≤5 years. However, in this age group, wheezing is highly heterogeneous, and not all wheezing indicates asthma ^(1)^. Therefore, it is difficult to distinguish bronchitis from asthma in children with acute wheezing, even if prospectively assessed. The difference in pathologies would result in different outcomes, including treatment response and final disposition. In this study, the predictive validity was assessed with stratification by age classes and body temperature, and the value was the same for children aged ≤5 years. Therefore, the MPIS could also assess symptoms of airway tract infection overlapping those of asthma because the six items of the MPIS, including the vital signs, are not specific to asthma and are related to the severity of respiratory effort. The other limitation lies in the reassessment period variability. The decision-making process for hospital admission may influence the results to some extent. However, these variations presuppose a clinical setting, and this study’s results may help interpret the MPIS’ utility in a real clinical setting. The complexity of the MPIS, which comprises four classes for six items (two stratified by age), may lead to random errors. In this study, this risk was managed by using an electronic scoring system for the MPIS, which automatically calculates the MPIS when the values for the six items and age are entered.

In summary, the MPIS showed good concurrent validity, predictive validity, and responsiveness in a wide clinical setting. Ten years after its introduction to our medical center, the MPIS has become popular among the staff and has proven efficacy as an asthma assessment tool in clinical practice. The MPIS helps physicians assess the severity of acute asthma more objectively, quantify the effectiveness of treatment, and predict outcomes. The objectivity of the score aids in the use of the MPIS as an outcome measure in clinical research in acute asthma care. The MPIS is recommended as an objective assessment tool for acute asthma exacerbation in children.

## Article Information

### Conflicts of Interest

None

### Acknowledgement

We thank all the residents, physicians, and nurses who participated in this study. Without their support, this study would not have been possible.

### Author Contributions

T. Maekawa contributed to the conception and design of this study, performed the statistical analyses and data management, and drafted the manuscript. Y. Ohya contributed to the concept of this study and gave advice on the interpretation of the study results. M. Mikami contributed to the statistical analysis of this study. S. Uematsu contributed to the implementation of this study. Ishiguro reviewed the manuscript and supervised the whole study process. All authors read and approved the final manuscript.

### Approval of the Institutional Ethics Committee

Approved Code: 679

Name of the Institution: National Center for Child Health and Development, Setagaya, Tokyo, Japan

Date of approval: 15 June 2011
